# The effect of basic public health service equalization on settlement intention of migrant workers in China: the mediating effect model based on subjective feelings

**DOI:** 10.1186/s13561-024-00534-2

**Published:** 2024-07-27

**Authors:** Pei Liu, Zhiping Long, Xuemeng Ding

**Affiliations:** 1 College of Economics and Management (China-Africa International Business School), Zhejiang Normal University, Jinhua, 321004 Zhejiang China; 2 School of Public Health, Harbin Medical University, Harbin, 150076 Heilongjiang China; 3 School of Humanities and Laws, Hebei University of Technology, Tianjin, 300131 China

**Keywords:** Basic public health services equalization, Migrant workers, Mediating effect model, Settlement intention

## Abstract

**Background:**

During the 14th Five-Year Plan, China aims to transform rural migrants into urban citizens and ensure equal access to public services to enhance new urbanization. Understanding migrant workers' settlement intentions is crucial for their citizenship development. Based on the fundamental role of the right to life and health, equalization of basic public health services is essential. Therefore, understanding the potential impact of public health services equalization on the settlement intention of migrant workers is crucial in China’s new urbanization.

**Method:**

In this study, we utilized data from the 2017 wave of China Migrants Dynamic Survey (CMDS) and employed the Propensity Score Matching method to investigate the impact of basic public health service equalization policy on the settlement intention of migrant workers. Additionally, we utilized the Mediation Effect Model to uncover the impact mechanism.

**Results:**

Our findings indicate that basic public health service equalization policy has a significant positive effect on increasing the settlement intention of migrant workers, with an even greater effect observed among the low-income group, the cross-provincial subsample, and the new generation subsample. The results of the Mediation Effect Model suggest that Basic public health service equalization policy can bolster the subjective integration willingness and subjective identity of migrant workers, thereby enhancing their settlement intention.

**Conclusion:**

Based on the results, we propose to strengthen the promotion of the basic public health service equalization policy and expand the coverage of health records to further increase the settlement intention of migrant workers.

## Background

The agricultural migrant population has emerged and expanded as a distinctive group in the context of China's economic and social development. Spanning all regions and sectors, these individuals have significantly contributed to China's industrialization and urbanization. The seventh national census of China revealed a staggering 375.82 million migrant workers, marking a 69.73 percent increase from 2010. However, the labor mobility in China shows an unstable nature [1], as migrant workers often refrain from permanent settlement, choosing instead to return to their places of origin [2]. Consequently, the settlement intentions of agricultural migrants have been the subject of extensive research, generating a substantial body of literature over the past two decades.

The settlement decision of the migrant workers is a rational choice influenced by a range of individual attributes. These include age, gender, marital status, education level, number of children, health status, job quality and stability, family migration, duration of residence, family income, and housing conditions [1, 3–12].

Settlement intention is not solely linked to individual and familial characteristics but is also closely tied to socio-economic and institutional factors [6]. Socio-economic factors play a pivotal role, with economic incentives such as employment and income [4, 13–15] being primary influencers, often correlated with the economic development level of the destination area. Numerous studies have demonstrated a preference among migrant workers for coastal cities, particularly the eastern coastal city clusters, due to their allure for settlement. Additionally, inland provincial capitals, cities with convenient transportation, resource endowments, and superior public services are also attractive settlement destinations for migrant workers [16, 17]. However, Chen & Wang [18] noted that while economic incentives significantly impact the probability of migrant workers' intention to settle in cities, the marginal effect diminishes over time.

In the realm of institutional factors, previous research has predominantly centered on the *hukou* system [1], which is considered to be the main or even the only reason why migrants cannot permanently settle in the destination city [19, 20]. For instance, Zhang & Wang's [21] study highlights that China's *hukou* system, which rigidly segregates residents into urban and rural categories, engenders significant disparities in access to social welfare, including education, social security, employment opportunities, and subsidized housing. Consequently, temporary rural migrants are barred from attaining urban citizenship and endure various forms of social exclusion and marginalization in China's urban areas [3, 6, 20]. As a result of institutional and social discrimination in urban areas, many individuals either return to their rural homes or maintain their transient status [22]. However, recent studies have indicated that the *hukou* system's significance in determining urban settlement for rural and urban migrants has been waning [23]. Particularly in recent years, China has vigorously advocated for the reform of the *hukou* registration system, gradually easing restrictions on household registration in numerous major cities [24]. This shift, by affording opportunities for urban and rural migrants to obtain official citizenship [17], mitigates the *hukou* system's impact on the settlement intentions of migrant workers.

Basic public services equalization is another institutional policy that profoundly influences the welfare of migrant workers. Since 2005, China has enacted the equalization policy for basic public services, encompassing education, employment, entrepreneurship, social insurance, medical and healthcare, social services, housing security, culture, sports, and more. This initiative aims to alleviate welfare disparities stemming from *hukou* distinctions and facilitate migrant workers' access to essential public services. Among these, the equalization policy for basic public health services garners the most attention, given the pivotal role of the right to health and the right to life.

However, research findings regarding the impact of this policy on the settlement intentions of migrant workers are inconsistent, and its underlying mechanism is seldom explored. For instance, Huang et al. [25] demonstrate that participation in urban social welfare programs enhances migrant workers' intention to reside in cities for an extended period and transition to urban household registration, with this influence growing as the migrant workers' duration of residence in the destination city increases. Meanwhile, Zheng et al. [26] discovered that social welfare, particularly health education, positively affects the settlement intentions of the migrant population in terms of economic advantage and air pollution. Similarly, Wu et al. [27] also observed that urban healthcare significantly enhances the settlement intentions of migrant workers. Even after accounting for individual characteristics, family traits, and migration specifics, the promotional effect of urban healthcare on settlement intentions remains substantial. Jia [28] asserted that establishing health records and providing access to health-related knowledge significantly and positively impacts migrants' intentions to permanently settle in the influx area by improving their health status and social integration. However, Lu [29] found that following the implementation of equalization of basic public health and medical services, the decline in social integration was the primary factor affecting the long-term urban settlement intentions of migrant workers. Consequently, the effectiveness of this policy, its impact on migrants' willingness to settle, and its underlying mechanisms will be the focus of an in-depth study in this paper.

The remainder of this paper is structured as follows: Methods section outlines the methodology employed in this study, while Results section presents the empirical findings. Discussion section provides a discussion of the results, including impact mechanism analysis, heterogeneity analysis and endogeneity discussion. Finally, Conclusions and implications section presents a summary of the conclusions, implications and limitations.

## Methods

### Data source

The data used in this study was sourced from the 2017 wave of the China Migrants Dynamic Survey (CMDS), a nationwide social follow-up survey conducted by the National Health Commission. The sampling frame of the CMDS encompassed the entire population of migrants aged 15 and above, who did not possess a local *hukou* (household registration), and had resided in their current locality for at least one month as of May 1, 2017. The 2017 CMDS employed a stratified multi-stage probability proportional to size sampling method. In total, the 2017 CMDS surveyed 169,989 individuals across 31 mainland provinces/autonomous regions/municipalities as well as the Xinjiang Production and Construction Corps. After excluding samples with missing personal income and specific public health service data, 139,831 observations were obtained.

### Variables and measures

#### Dependent variables

The dependent variable in this study is the settlement intention of the respondents, which is a dichotomous variable. In the questionnaire, respondents were asked "Do you plan to settle here for some time to come?”. A positive answer was coded as 1, while a negative answer was coded as 0.

#### Independent Variables

In 2009, Chinese government introduced Basic Public Health Service (BPHS) as a landmark health policy to facilitate universal access to health services, aiming to provide free basic health services to all citizens. The initial package in 2009 included 9 categories, such as the establishment of health records, health education, child immunization, and more [30]. By 2022, the service system had been expanded to 22.

The independent variable in this study is whether the respondent has a health record, which is a dichotomous variable. In the questionnaire, respondents were asked whether a local health record had been established. A positive answer was coded as 1, while a negative answer was coded as 0. The basis for the selection of substitute variables is as follows:

First of all, encouraging residents to establish health records is a crucial focus for medical and health departments at all levels when implementing of basic public health services equalization policy. As per the design of national basic public health services, funds are allocated by governments according to a certain amount per capita, providing an incentive for local governments to promote the establishment of health records among residents. Consequently, the proportion of established health records can significantly indicate the level of equalization of local basic public health services.

Second, the establishment of health records is the initial requirement for migrants to access local public health services. Prior to the implementation of the equalization policy of basic public health services, access to these services was closely linked to *hukou* registration, with local *hukou* registration being a prerequisite to enjoy local public health services. The aim of the equalization policy is to address the inequality stemming from such *hukou* registration restrictions. However, in practice, certain procedures are still necessary, such as migrants needing to establish local health records to access the same medical and other public health services as local residents. Therefore, possessing a health record signifies that migrant are eligible for local public health services, indicating that they benefit from basic public health services equalization.

#### Mediating variables

The mediating variables are the subjective local identity and the subjective integration willingness of the respondents, both of which are ordinal variables. Respondents were asked about their recognition of "I am willing to integrate into the natives and become one of them" and "I think I am already a native." The optional answers ranged from 1 to 4, with higher values indicating stronger subjective integration intention and local identity.

#### Control variables

Control variables include individual and family characteristic variables. Individual characteristics encompass age, gender, marital status, education level, health status, personal income, years of residence in the city, mobility range, and the number of cities previously lived in. Family characteristics include family size and per capita monthly income. Additionally, regional dummy variables were included to account for regional differences. Table 1 provides an overview of the variable selection and definitions.

**Table 1:** Variables description

Category	Variable	Definition
Explained Variable	Settle	Intend to settle here = 1, otherwise = 0
Core Explanatory Variables	HR	Own a health record = 1, otherwise = 0
Mediating Variables	Sub_ identity	Range form 1–4, the higher the score, the stronger the feeling of identifying being a native
	Sub_integration	Range form 1–4, the higher the score, the stronger the subjective willingness to integrate with natives
**Control Variables**		
Personal Variables	Age	Age > = 15
	Gender	Male = 1, female = 0
	Marriage	Married = 1, otherwise = 0
	Healthy	Healthy = 1, basically healthy = 2, unhealthy but able to take care of himself = 3, unable to take care of himself = 4
	Education	Illiterate = 1, primary = 2 school, middle school = 3, High school/technical secondary school = 4, junior college = 5, undergraduate = 6, graduate = 7
	Years	Number of years since working here
	Range	Cross province = 1, cross city within province = 2, cross county within city = 3
	Cities	Number of cities that have worked before
	Pincome	Monthly personal income
Family Variables	Familysize	Number of family members
	Income_per	Average monthly household income per capita in the past year
Regional Variable	Province	East = 1, central and west = 0

To address the heteroscedasticity arising from cross-sectional data, individual monthly income and household per capita monthly income were logarithmically transformed during the empirical analysis.

### Descriptive statistics

As shown in Table 2, 82.49% of migrant workers were willing to settle down in the whole sample, only 26.73% of migrant workers had health records. The subjective identity was at the average level and the subjective integration willingness was high. From the perspective of personal characteristic variables, the average age of the interviewees was 35.96 years old, the proportion of males was 57.08%, the proportion of married was 81.06%, the average education level was middle and high school level, the average health condition was good, the average monthly income was 4,328.04 yuan, the average number of years to move to the local was 6.26 years, most of them were inter-provincial or inter-municipal flow. The average number of cities that had previously moved was 2.03. From the perspective of family characteristic variables, the average family size was 3.11, and the average monthly income of per capita family was 2651.52 yuan. The sample of those who migrated to eastern provinces accounted for 45.43%.

**Table 2:** Descriptive statistics

Variable	Full		Treatment		Control		Mean Diff
	Mean	Std. Dev	Mean	Std. Dev	Mean	Std. Dev	
Settle	0.8249	0.3800	0.8610	0.3460	0.8118	0.3909	-0.049***
HR	0.2673	0.4426	1	0	0	0	-
Sub_identity	2.9525	0.7626	3.1176	0.7406	2.8922	0.7616	-0.225***
Sub_integration	3.3188	0.6336	3.4237	0.6166	3.2805	0.6353	-0.143***
Age	35.9629	9.8409	36.3427	9.5348	35.8244	9.9467	-0.518***
Gender	0.5708	0.4950	0.5549	0.4970	0.5766	0.4941	0.022***
Marriage	0.8106	0.3918	0.8488	0.3582	0.7966	0.4025	-0.052***
Education	3.4930	1.1578	3.5736	1.1564	3.4637	1.1569	-0.110***
Healthy	1.1678	0.4106	1.1528	0.3930	1.1732	0.4167	0.020***
Pincome	4328.0450	3869.5960	4193.7590	3444.6720	4377.0390	4012.3380	183.280***
Year	6.2688	5.9933	6.8229	5.8157	6.0666	6.0442	-0.756***
Range	1.6674	0.7516	1.7875	0.7632	1.6236	0.7425	-0.164***
Cities	2.0371	1.9626	1.9079	1.6932	2.0842	2.0501	0.176***
Familysize	3.1108	1.2031	3.1994	1.1409	3.0785	1.2235	-0.121***
Income_per	2651.5210	2217.2900	2503.5470	1977.8490	2705.5080	2296.0830	201.960***
Province	0.4543	0.4979	0.3665	0.4819	0.4864	0.4998	0.120***
N	139,831		115,350		24,481		-

^*^P < 0.10

^**^P < 0.05

^***^P < 0.01

Based on whether the respondents had health records, the samples were categorized into the control group and the treatment group. Analysis revealed a notable selection bias among the samples with health records. Specifically, at the 1% significance level, migrant workers with health records exhibited a 4.9% higher settlement intention compared to those without health records. Additionally, they displayed higher levels of subjective identity and subjective integration willingness. When comparing control variables, it was found that at the 1% significance level, the average age, proportion of married individuals, education level, years since working here, and family size of the samples with health records were higher than those without health records. Conversely, the proportion of males, health status, monthly personal income, number of cities moved, and monthly family income per capita were lower among those with health records.

### Empirical model

To effectively measure the effect of basic public health services equalization on the settlement intention of migrant workers, the equation is defined as:

1 $$\mathit{Pr}(Settle=1)=\Phi (\alpha +\beta H{R}_{i}+\gamma {X}_{i}+{\varepsilon }_{i})$$

where, $Settle_{i}$ denotes the settlement intention of the individual $i$. $X{}_{i}$ represents the vector of control variables. $HR_{i}$ represents a binary variable of whether the respondent $i$ has a health record. $\beta$ is the parameter to be estimated, $\varepsilon_{i}$ is the disturbance term. Considering that the selection problem may exists, that is the migrant workers decide whether to establish health records by themselves, if we ignore the self-selection problem and directly estimate Eq. (1), the estimation results would be biased.

In this paper, Propensity Score Matching (PSM) was used to construct a counterfactual framework to achieve an approximate random assignment to overcome the selection problem. The logic of the PSM method involves selecting or creating a control group for each treatment group from a sample of migrant workers without health records via matching, ensuring that the two groups are similar in characteristics except for their health records. This allows the outcome variables of the two groups to be considered as the results of the same individual in two different experiments, with the difference in outcome variables representing the net effect of having health records, that is the counterfactual analysis framework defined by Rosenbaum et al. [31].

The Propensity Score Matching yields two results: the ATT (average effect of settlement intention for those who own health records) and ATE (average effect of settlement intention for those who own health records and those who don’t). Given the focus of this study on the effect of owning a health record on settlement intention, the ATT is the key observation.

The steps to calculate the average treatment effect (ATT) through PSM are as follows:

1. Select covariable $X_{i}$.We have added 12 covariables such as individual level, family level and regional dummy variables that may affect the relationship according to existing relevant literature to ensure that the negligible assumption is satisfied.
2. Estimate the probability of an individual establishing a health record:

2 $$PS(X_{i} ) = \Pr (HR = 1|X_{i} ) = E(HR|X_{i} )$$

where, $X_{i}$ is the factors that affect migrant workers to establish health records, $PS$ is the probability of individuals to establish health records, that is, Propensity Score. According to the above regression equation, the propensity score of each individual to establish a health record can be calculated as the basis for matching.

(3) Based on the matched samples, compare the difference of settlement intention between the group with and without a health record, so as to find the causality coefficient of establishing a health record on settlement intention, that is, Average Treatment Effected on Treated. The specific calculation formula is set as followed:

3 $$\begin{gathered} ATT = E[(Settle_{1i} - Settle_{0i} )|HR_{i} = 1] \hfill \\ = E\{ E[Settle_{1i} - Settle_{0i} ]|HR_{i} = 1,PS(X_{i} )\} \hfill \\ = E\{ E[Settle_{1i} |HR_{i} = 1,PS(X_{i} )]\} - E\{ E[Settle_{0i} |HR_{i} = 0,PS(X_{i} )|HR_{i} = 1]\} \hfill \\ \end{gathered}$$

where, $Settle_{1i}$,$Settle_{0i}$ represent the settlement intention of individuals with health records and individuals without health records respectively. $PS \, \left( {X_{i} } \right)$ is a continuous variable and can be analyzed using nearest neighbor matching, radius matching, and kernel matching.

(4) Finally, the matching quality is analyzed. PSM requires that the propensity scores of the treatment and control groups have sufficient areas of overlap, that is, common support assumption.

Further, this paper introduces two mediating variables (Sub_integration and Sub_identity) and Mediation Effect Model is used to test the mechanism of the effect of public health services equalization on the settlement intention of migrant workers. The model is set as follows:

4 $$Med_{i} = \phi_{{0}} { + }\phi_{{1}} HR_{i} + \phi_{2} X + \varepsilon_{i2}$$

5 $$Y_{i} = \theta_{{0}} { + }\theta_{{1}} HR_{i} + \theta_{2} Med_{i} + \theta_{3} X + \varepsilon_{i3}$$

where, $Med_{i}$ represents the mediation variable, and the verification mainly consists of three steps. In the first step, test $\phi_{1}$ and $\theta_{{2}}$ in turn, if both coefficients are significant, then the mediating effect exists. Second, if $\theta_{1}$ is not significant, the mediating effect is complete, and the independent variable ($HR_{i}$) can affect the dependent variable ($Settle_{i}$) only through the mediating variable ($Med_{i}$). If $\theta_{1}$ is significant, the mediating effect is partial, and independent variables affect dependent variables partly through mediating variables. In the third step, if one of $\phi_{1}$ and $\theta_{{2}}$ is not significant, the Sobel test is conducted, if the significance test is passed, the mediating effect is partial, otherwise, there is no mediating effect.

## Results

### Baseline regression results

The baseline regression results using Probit model are presented in Table 3. In column (1), the estimates without any control variables indicate that migrant workers with health records are 5.14% more likely to settle down than those without health records at the 1% significance level. In column (2), after incorporating personal characteristic control variables, the coefficient decreases to 3.59%. Specifically, at the 1% significance level, the likelihood of settling down decreases by 0.15% with each additional year of age, and married migrant workers exhibit an 8.26% higher intention to settle down compared to unmarried migrant workers. Additionally, higher education levels, better health status, higher monthly income, and closer migration ranges are associated with a higher likelihood of settling. Each additional year of working in the local area is linked to a 0.65% increase in the intention to settle, while an increase of 1 in the number of flowing cities is associated with a 0.35% increase in settlement intention. Upon incorporating family characteristic control variables, the results in column (3) show that at the 1% significance level, higher per capita annual family income is associated with a stronger settlement intention among migrant workers. Additionally, for each additional family member, the settlement intention increases by 1.88%. The regression results for other variables are largely consistent with those in column (2), with slight variations in values. Finally, in column (4), the regression results of the province dummy variable indicate that, holding other variables constant, migrant workers in eastern provinces are more likely to settle down.

**Table 3:** Baseline regression results

	(1)	(2)	(3)	(4)
Variables	Settle	Settle	Settle	Settle
HR	0.0514***	0.0359***	0.0355***	0.0384***
	(0.0024)	(0.0024)	(0.0023)	(0.0024)
Age		-0.0015***	-0.0012***	-0.0011***
		(0.0001)	(0.0001)	(0.0001)
Gender		-0.0022	-0.0012	-0.0004
		(0.0021)	(0.0021)	(0.0021)
Marriage		0.0826***	0.0616***	0.0602***
		(0.0027)	(0.0032)	(0.0032)
Education		0.0342***	0.0317***	0.0316***
		(0.0010)	(0.0010)	(0.0010)
Healthy		-0.0276***	-0.0252***	-0.0239***
		(0.0024)	(0.0024)	(0.0024)
Pincome		0.0082***	0.0031***	0.0025***
		(0.0007)	(0.0008)	(0.0008)
Year		0.0065***	0.0061***	0.0061***
		(0.0002)	(0.0002)	(0.0002)
Range		0.0150***	0.0179***	0.0252***
		(0.0014)	(0.0014)	(0.0015)
Cities		0.0035***	0.0026***	0.0026***
		(0.0006)	(0.0006)	(0.0006)
Familysize			0.0188***	0.0187***
			(0.0011)	(0.0011)
Income_per			0.0324***	0.0298***
			(0.0019)	(0.0018)
Province				0.0328***
				(0.0022)
N	139,831	139,831	139,831	139,831

①*P < 0.10,**P < 0.05,***P < 0.01

②The coefficients reported in the table are marginal effects

③The value in brackets is Delta-method standard error

### Propensity score matching estimation results

Due to the self-selectivity of the independent variable, the estimation results of Probit are biased. Therefore, the propensity score matching method is utilized for re-estimation. To ensure the reliability of the estimation results, various matching methods such as Nearest Neighbor Matching, Radius Matching, and Kernel Matching are typically employed for comparison.

As shown in Table 4, the T values of the ATT corresponding to Nearest Neighbor Matching (1-to-1), Nearest Neighbor Matching (1-to-4), Radius Matching, and Kernel Matching are 12.18, 15.54, 12.18, and 17.54, respectively. All of these values exceed the T value corresponding to a 1% significance level of 2.76. This indicates that, after controlling for sample selectivity bias, owning a health record has a positive effect on the settlement intention of migrant workers. Specifically, migrant workers with a health record were 3.8% more likely to settle down than those without a health record.

**Table 4:** PSM results

Matched Method	ATT	S.E	T Value	Mean ATT
1 to 1 Neighbor Matching	0.0378	0.0031	12.18	0.0382
1 to 4 Neighbor Matching	0.0384	0.0025	15.54	
Radius Matching	0.0378	0.0031	12.18	
Kernel Matching	0.0386	0.0022	17.54	

#### Matching quality test

**Fig. 1:**
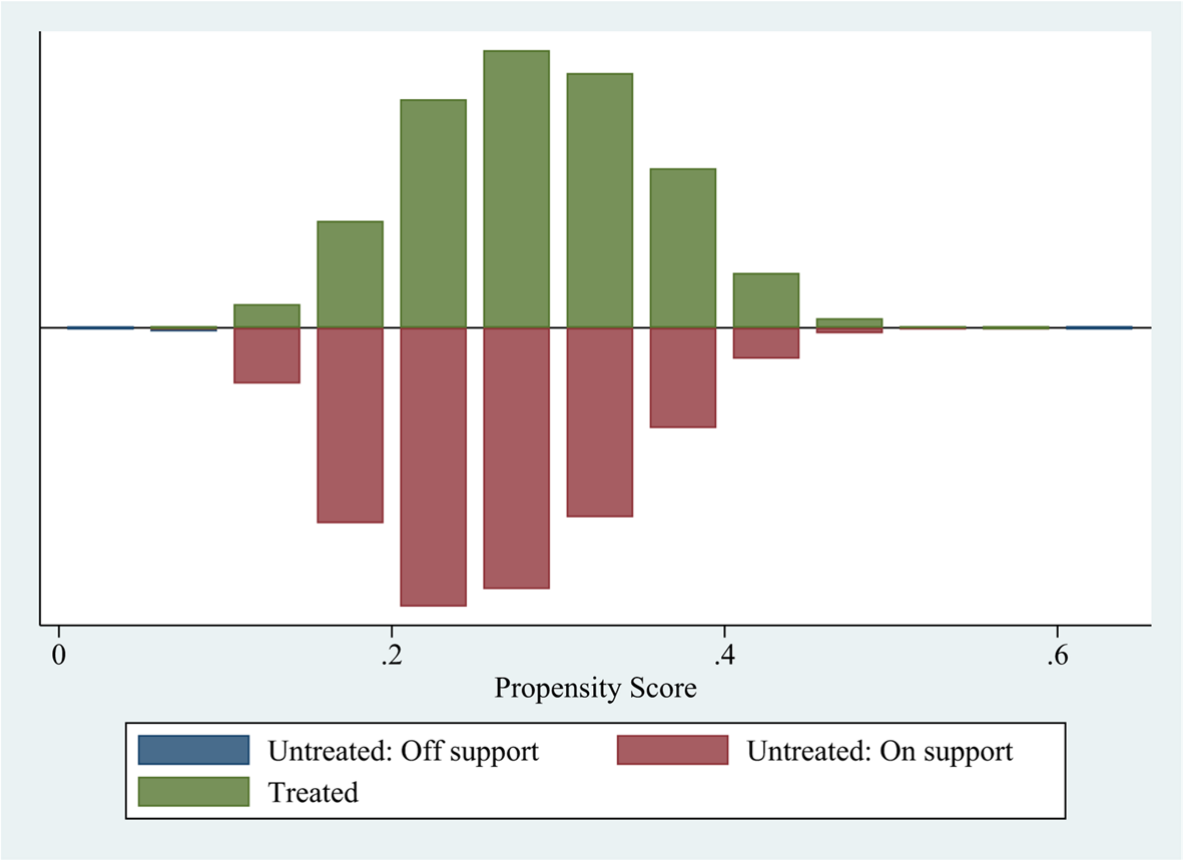
Common range of propensity score values

After calculating the propensity matching score, it is essential to test the common support hypothesis to ensure the effectiveness and quality of the matching. The common support domain refers to the overlapping interval between the samples of the treatment group and the control group. If the common support domain is too narrow, it can lead to ineffective matching and a lack of samples outside this range. There are three methods to test the common support hypothesis: common support domain bar graph, empirical density function graph, and ROC curve. This paper primarily presents the results of Nearest Neighbor (1–4) Matching estimates from the bar graph of the common support domain (See Fig. 1 and Table 5).

**Table 5:** The common range of supported domains

Treatment assignment	Off support	On support	Total
Untreated	21	102,431	102,452
Treated	0	37,379	37,379
Total	21	139,810	139,831

As depicted in Fig. 1, the common support interval between the group that owns a health record and the group that does not own a health record was relatively large, indicating favorable data application conditions.

Table 5 displays the common range of supported domains for propensity score matching. Among the 139,831 observed samples, 21 samples in the control group were not within the common value range, while the remaining 139,810 samples were within this range.

Another hypothesis of the PSM method is the balance hypothesis, which aims to balance the distribution of explanatory variables between the treatment group and the control group. After matching, if the standardized deviation between the treatment group and the control group is less than 20%, it indicates a successful matching. Table 6 presents the results of the balance test. Following sample matching, the standardized deviation of explanatory variables was reduced to 0% to 0.5%, effectively reducing the overall bias. The P-value of the likelihood ratio test indicated that the joint significance test of owning a health record was significant at the 1% level before matching, but was rejected after matching. Additionally, the Pseudo-R2 values decreased significantly. In conclusion, the sample matching was successful, and the propensity score estimation was effective.

**Table 6:** Balance test results

Variable	Sample	Mean		Standardized bias (%)	Bias reduction (%)	T test	
		Treated	Control			T Value	*P* Value
Age	U	36.343	35.824	5.3		8.72	0.000
	M	36.343	36.27	0.7	86	1.03	0.302
Gender	U	0.55488	0.5766	-4.4		-7.27	0.000
	M	0.55488	0.5524	0.5	88.4	0.7	0.486
Marriage	U	0.84885	0.7967	13.7		22.08	0.000
	M	0.84885	0.84792	0.2	98.2	0.35	0.726
Education	U	3.5736	3.4637	9.5		15.72	0.000
	M	3.5736	3.5668	0.6	93.8	0.79	0.428
Healthy	U	1.1528	1.1732	-5		-8.24	0.000
	M	1.1528	1.1513	0.4	92.7	0.52	0.601
Pincome	U	8.0579	8.0655	-0.6		-0.97	0.333
	M	8.0579	8.0545	0.3	54.2	0.37	0.709
Year	U	6.8229	6.0666	12.8		20.92	0.000
	M	6.8229	6.8245	0	99.8	-0.04	0.972
Range	U	1.7875	1.6236	21.8		36.25	0.000
	M	1.7875	1.7852	0.3	98.6	0.4	0.690
Cities	U	1.9079	2.0842	-9.4		-14.88	0.000
	M	1.9079	1.906	0.1	98.9	0.16	0.872
Familysize	U	3.1994	3.0785	10.2		16.65	0.000
	M	3.1994	3.1988	0.1	99.5	0.07	0.944
Income_per	U	7.6233	7.6685	-6.2		-10.05	0.000
	M	7.6233	7.6202	0.4	93.2	0.58	0.562
Province	U	0.3665	0.4864	-24.4		-40.08	0.000
	M	0.3665	0.3638	0.5	97.8	0.75	0.451

##### Robustness test

To ensure the reliability of PSM results, this paper employs two methods to test the robustness. Firstly, the sample size was reduced, and the study subjects were limited to those under 60 years old. A total of 137,809 samples were obtained, and PSM was re-estimated. The results are presented in Table 7, indicating that even when the sample is limited to those under 60 years old, the migrant workers with health records still exhibits a significant difference compared to the sample without health records, with a marginal impact of approximately 3.74%.

**Table 7:** PSM results (subsample under 60 years old)

Method	ATT	S. E	T Value	Mean ATT
1 to 1 Neighbor Matching	0.0374	0.0031	12.01	0.0374
1 to 4 Neighbor Matching	0.0364	0.0025	14.69	
Radius Matching	0.0374	0.0031	12.01	
Kernel Matching	0.0384	0.0022	17.30	

The second robustness test method is to change the substitute variable. In the CMDS questionnaire, respondents were queried about the various types of health education they had received. As a result, the number of types of health education received by the respondents was utilized as a proxy variable in this study to reflect the fundamental public health services accessed by migrant workers. The results are presented in Table 8. The findings indicated that the implementation of health records had a significant impact on the increase in the number of migrant workers receiving health education. This outcome serves to validate the reliability of the aforementioned empirical results.

**Table 8:** PSM results (replacing the explanatory variable)

Method	ATT	S. E	T Value	Mean ATT
1 to 1 Neighbor Matching	0.0133	0.0055	2.42	0.0129
1 to 4 Neighbor Matching	0.0118	0.0044	2.68	
Radius Matching	0.0133	0.0031	2.42	
Kernel Matching	0.0131	0.0039	3.33	

## Discussion

### Impact mechanism analyses

The decision to settle is not solely determined by economic factors but is also influenced by psychological factors. Many researches have highlighted the significance of identity and attitude towards the city as crucial factors impacting settlement intentions [10, 32]. Chavez [33] explored the importance of "imaginary communities" on the willingness of undocumented migrants to remain in the United States, finding that imagining oneself as part of a local community had a powerful effect on settlement, emphasizing the importance of belonging.

Belonging is defined as "the feeling, belief, and expectation that one fits in and takes a place in a group, a feeling of acceptance by the group, and a willingness to sacrifice for the group" [34]. Migrants often encounter social exclusion and a lack of belonging, leading to isolation and social exclusion, which can negatively impact their mental, social, and physical health [35–38). The public resources accessible to individuals play a pivotal role in shaping their sense of belonging. Chien [39] notes that when category members display group favoritism in terms of resource allocation or evaluation, they tend to report a higher sense of belonging. In the context of the equalization of basic public services in China, migrant workers can access the same public resources, potentially enhancing their sense of belonging and subsequently affecting their settlement intention.

Thus, we sought to explore the impact mechanism behind the influence of owning a health record on settlement intention and the results are displayed in Table 9. Column (1) reports the basic result of the Probit model without adding any mediating variable, showing that the influence of having a health record on settlement intention is significantly positive at the 1% level. Column (2) demonstrates the impact of health records on subjective local identity, revealing that migrant workers with health records exhibit a stronger sense of being local at the 1% significance level.

**Table 9:** Test results of mediating effect model

	Settlement intention	Subjective Identity Effect		Subjective Integration Effect	
	(1)	(2)	(3)	(4)	(5)
VARIABLES	Settle	Sub_identity	Settle	Sub_integration	Settle
HR	0.0384***	0.4081***	0.0293***	0.3583***	0.0269***
	(0.0024)	(0.0119)	(0.0023)	(0.0122)	(0.0023)
Sub_identity			0.0609***		
			(0.0013)		
Sub_integration					0.1025***
					(0.0015)
Control	Yes	Yes	Yes	Yes	Yes
Pseudo R2	0.0405	0.0496	0.0562	0.0269	0.0752
N	139,831	139,831	139,831	139,831	139,831

The value in brackets is standard error

Column (4) reports the effect of health records on subjective integration willingness, indicating that migrant workers with health records have a stronger subjective integration willingness at the 1% significance level. Furthermore, Column (3) and Column (5) present the estimated result of adding the mediating variables and the core explanatory variable into the model simultaneously, revealing that the mediating effect of subjective identity and subjective integration willingness exists.

In conclusion, possessing a health record can enhance the subjective identity and subjective integration willingness of migrant workers, thereby improving their settlement intention.

### Heterogeneity analysis

#### Income heterogeneity

Numerous studies have confirmed the importance of economic incentives in migrant population's intention to settle. However, is there heterogeneity of economic incentive factors in the effect of basic public health services equalization on the settlement intention of migrant workers? This paper divides the sample into four equal parts based on income level and repeats the PSM estimate.

The results, as shown in Table 10, demonstrate that basic public health services equalization policy has a significant promoting effect on the settlement intention of migrant workers with different income levels. Among them, the promotion effect on the settlement intention of low-income individuals is the greatest. The higher the income level, the less marginal effect. The possible explanation is that the high-income individuals can afford basic public health services themselves or obtain services from other sources. However, the lower the income of the migrant workers, the more they rely on free public health services. Therefore, the equalization policy for basic public health services may have a greater positive impact on low-income individuals.

**Table 10:** PSM results (Income heterogeneity)

Method	Low income		Mid-low income		Mid-high income		High income	
	ATT	T Value	ATT	T Value	ATT	T Value	ATT	T Value
1 to 1 Neighbor Matching	0.0659 (0.0154)	4.27	0.0438 (0.0106)	4.13	0.0411 (0.0064)	6.33	0.0385 (0.0038)	10.26
1 to 4 Neighbor Matching	0.0605 (0.0121)	4.97	0.0490 (0.0084)	5.79	0.0437 (0.0052)	8.43	0.0324 (0.0030)	10.95
Radius Matching	0.0659 (0.0154)	4.27	0.0438 (0.0106)	4.13	0.0411 (0.0064)	6.33	0.0385 (0.0038)	10.26
Kernel Matching	0.0499 (0.0108)	4.61	0.0527 (0.0075)	6.99	0.0478 (0.0046)	10.35	0.0307 (0.0026)	11.57

The value in brackets is standard error

#### Heterogeneity of flow range

Furthermore, this paper divides migrant workers into inter-provincial, intra-provincial, and intra-city migrant workers according to their flow range and repeats PSM estimation. The results in Table 11 show that when migrant workers are divided into three groups based on their flow range, the impact of having a health record on their settlement intention increases with the greater flow range.

**Table 11:** PSM results (flow range heterogeneity)

	cross-province		cross-city		cross-county	
Method	ATT	T Value	ATT	T Value	ATT	T Value
1 to 1 Neighbor Matching	0.0398 (0.0048)	8.25	0.0397 (0.0050)	7.88	0.0277 (0.0068)	4.08
1 to 4 Neighbor Matching	0.0423 (0.0037)	11.08	0.0399 (0.0040)	9.92	0.0269 (0.0055)	4.91
Radius Matching	0.0397 (0.0048)	8.25	0.0397 (0.0050)	7.88	0.0277 (0.0068)	4.08
Kernel Matching	0.0411 (0.0034)	11.41	0.0411 (0.0036)	12.12	0.0308 (0.0049)	6.24

The value in brackets is standard error

The possible explanation is that the relative income gap between the workplace and their hometown acts as a positive factor that attracts migrants to settle in their workplace, while social exclusion and other workplace-related factors serve as negative influences on their settlement intentions. Migrant workers with larger flow range may prioritize the improvement in relative income resulting from migration and may pay less attention to negative factors such as social exclusion. Besides, cross-provincial migrants generally move to places with high economic development level, where public health resources are better than those in their hometown. Therefore, after the implementation of basic public health services equalization policy, immigrants can obtain better public health services in their workplace than those in their hometown, which enhances their willingness to settle down.

#### Inter-generational heterogeneity

Migrant workers of varying ages have distinct requirements for public health services, therefore the influence of health records on settlement intention may vary across generations. As per the National Bureau of Statistics' definition, the new generation comprises individuals born after 1980, while those born before this year are classified as the older generation. The regression results in Table 12 indicate that the equalization of basic public health services has a greater effect on the settlement intention of the new generation than on the older generation. The potential explanation is that the younger generation has weaker attachment to their hometown, and if they can access the same basic public health services as their hometown, their hometown becomes less attractive, leading to a stronger willingness to settle down.

**Table 12:** PSM results (Inter-generational heterogeneity)

Method	The new generation		The older generation	
	ATT	T Value	ATT	T Value
1 to 1 Neighbor Matching	0.0459 (0.0039)	11.65	0.0326 (0.0050)	6.47
1 to 4 Neighbor Matching	0.0409 (0.0031)	13.11	0.0345 (0.0040)	8.61
Radius Matching	0.0459 (0.0039)	11.65	0.0326 (0.0050)	6.47
Kernel Matching	0.0401 (0.0028)	14.46	0.0358 (0.0036)	9.95

The value in brackets is standard error

#### Discussion of endogeneity

The presence of missing variables, reverse causality, and selection bias can lead to endogenous problems, ultimately resulting in estimation errors. In an effort to mitigate the impact of missing variables, this paper references existing studies and endeavors to incorporate individual, family, and regional characteristics as control variables, aiming to minimize estimation errors arising from the absence of key variables. To address potential selection bias, the paper adopts the Propensity Score Matching (PSM) approach. This section specifically delves into the endogeneity issues stemming from reverse causality. For instance, individuals who are inclined to settle down may be more likely to invest time in establishing a comprehensive health record, while those who are not inclined to settle may not prioritize developing such a record. We employ instrumental variable method and normative analysis to examine the reverse causality problem.

According to Zhao (2022) [40], the financial transparency of Chinese municipal governments in 2017 was utilized as an instrumental variable, which sources comes from the annual report conducted by Tsinghua University since 2012 and is widely used in China-focused research studies [41]. Government transparency refers to the openness of the decision-making process and relevant government information, which also ensures the opportunity for citizens to obtain public information. Government information openness and transparency is a prerequisite for ensuring the reasonable allocation of information resources [42].

Instrumental variable must satisfy two key requirements: correlation and externality. Firstly, correlation refers to the strong association between fiscal transparency and the level of equality in basic public services. Theoretically, enhancing the efficiency and fairness of financial fund allocation for basic public services through appropriate institutional arrangements can foster the equalization of these services. The disclosure of government financial information empowers the public to effectively oversee the revenue and expenditure activities of local governments, thereby incentivizing them to optimize the allocation of financial resources and allocate more funds to basic public services This theoretical hypothesis is supported by empirical data, improving financial transparency can improve the quality of public services [43].The second requirement is exogeneity, fiscal transparency involves the comprehensive and timely disclosure of government structure and functions, fiscal policy trends, public sector accounts, and fiscal planning information to the public. However, as migrant workers do not directly receive financial transfer payments from local governments, fiscal transparency does not have a direct impact on the establishment of health records for migrants.

Table 13 presents the results of the two-stage least squares (2SLS) estimates using the instrumental variables. The first stage results demonstrate that higher financial transparency is associated with a greater level of basic public services equalization, signifying a significant positive correlation between the two, meeting the correlation requirements of instrumental variables. Besides, the F-value of the first stage is significantly higher than 10, indicating that it can be considered a robust instrumental variable. The regression results of the second stage indicate that the marginal effect is significant at the 1% level, consistent with previous findings.

**Table 13:** IV estimation results

Variables	First Stage	Second Stage
HR		0.0492***(11.71)
Financial Transparency	0.0018***(23.70)	
Control F Value	yes 314.28	yes
Obs	129,102	129,102

①*P < 0.10, **P < 0.05, ***P < 0.01

②Figures in brackets are t-statistics value

Again, the instrumental variable estimation results further confirms that basic public health services equalization policy has a positive promoting effect on settlement intention of migrant workers.

Although, there are some people who decide to settle down before setting up a health record, but this way of thinking doesn't work for everyone. Imagine that there are two regions, one of which has a higher degree of equality of basic public health services in region A than region B. Under the same conditions, it is obvious that the migrant workers will be more willing to settle in region A rather than B, which seems more logical. Besides, for the following reasons, we believe that basic public health services equalization policy is more likely to promote the migrant population's willingness to settle, rather than the reverse relationship.

First of all, for Chinese migrant workers, they leave their hometown to obtain higher economic income, public resources and better welfare, which is a fundamental truth that aligns with the national conditions of China. And the public health services available after the establishment of a health record are clearly a public resource that can enhance their welfare.

Secondly, after the implementation of basic public health services equalization policy, migrant workers can establish health records and enjoy local medical resources and public health services as natives, then improve the quality of life, and thus increase their willingness to settle down. In combination with the mechanism of action, the establishment of health records can enable migrant workers to obtain the same public health services as local residents, improve the sense of local identity and integration, and thus enhance the intention to settle.

## Conclusions and implications

### Conclusions

In our artical, we examined the effect of owning a health record on settlement intentions of migrant workers using 2017 wave of CMDS. Throughout the analysis, we primarily employed PSM to uncover the net effect of possessing a health record on settlement intention. The findings indicated that owning a health record indeed increased settlement intentions of migrant workers, with a more pronounced effect observed in low-income groups, the inter-provincial subsample and the new generation subsample.

As the economic status of the migrant population improves, factors such as career stability and social inclusion have become increasingly crucial in settlement decision-making. This paper utilized the Mediation Effect Model to explore the mechanisms through which owning a health record affects settlement intentions, revealing that it enhances migrants' sense of belonging and their willingness to integrate into their new communities. These mechanisms underscore the significance of considering psychological and social factors in the settlement decisions of migrant workers.

### Implications

These findings bear substantial policy implications. Firstly, Chinese government should intensify efforts to promote equal access to basic public services, thereby bolstering migrant workers' willingness to settle by expanding the coverage of basic public health services. This necessitates heightened publicity campaigns to encourage migrant workers to establish health records. Secondly, basic public health services equalization policy should be targeted towards vulnerable groups, particularly low-income individuals and families.

The contributions of this article are threefold. Firstly, it offers new empirical evidence on the effect of equal access to basic public health services on settlement intentions of migrant workers, based on a large-scale national micro-tracking survey. Secondly, by employing PSM to address confounding variables, the study provides a more accurate estimation of the net effect of policy impacts, overcoming issues related to sample self-selection. Lastly, by integrating psychology and sociology, this paper delves into the influence mechanism of equalization policy on settlement intentions, offering valuable insights for the formulation of relevant policies.

### Limitations

This study has several limitations that should be acknowledged. Firstly, despite the extensive efforts made in this paper, such as incorporating theoretical arguments, testing competing explanations, and endeavoring to minimize the estimation error resulting from endogeneity issues to enhance the credibility of the empirical results, it is important to acknowledge that there may still be various confounding factors influencing the settlement intention of the migrant workers that have not been observed. Secondly, the variations in the quality of public health services across different regions may have diverse impacts on the subjective feelings and settlement intentions of migrant workers. Additionally, control variables such as urbanization index and urban convenience are critical factors influencing the settlement intentions of migrant workers. While this study utilized the method of controlling provincial dummy variables to address differences in urban characteristics, the accuracy of the adjustments may be limited due to data constraints. Future research from our group aims to address these limitations by incorporating more comprehensive data.

## Data Availability

Publicly available datasets were analyzed in this study. This data can be found at: https://chinaldrk.org.cn/wjw/#/home.
