# Correction: The effect of basic public health service equalization on settlement intention of migrant workers in China: the mediating effect model based on subjective feelings

**DOI:** 10.1186/s13561-025-00593-z

**Published:** 2025-02-12

**Authors:** Pei Liu, Zhiping Long, Xuemeng Ding

**Affiliations:** 1 College of Economics and Management (China-Africa International Business School), Zhejiang Normal University, Jinhua, 321004 Zhejiang China; 2 School of Public Health, Harbin Medical University, Harbin, 150076 Heilongjiang China; 3 School of Humanities and Laws, Hebei University of Technology, Tianjin, 300131 China

**Correction:** ***Health Econ Rev*** **14, 58 (2024)**

10.1186/s13561-024-00534-2

Following the publication of the original article [1], the statement in the Funding information section was incorrectly given as ‘Thanks to the Migrant Population Service Center, National Health Commission of China for the data support’ and should have read ‘This work was supported by Zhejiang Federation of Humanities and Social Sciences Circles(2023B006) awarded to Pei Liu’.

The original article [1] has been updated.
